# The HP0256 gene product is involved in motility and cell envelope architecture of *Helicobacter pylori*

**DOI:** 10.1186/1471-2180-10-106

**Published:** 2010-04-08

**Authors:** François P Douillard, Kieran A Ryan, Michael C Lane, Delphine L Caly, Stanley A Moore, Charles W Penn, Jason Hinds, Paul W O'Toole

**Affiliations:** 1 Department of Microbiology & Alimentary Pharmabiotic Centre, University College Cork, Ireland; 2 Department of Biochemistry, University of Saskatchewan, Saskatoon, Saskatchewan, Canada; 3 School of Biosciences, University of Birmingham, Edgbaston, Birmingham, UK; 4 Bacterial Microarray Group, Division of Cellular and Molecular Medicine, St George's University of London, Cranmer Terrace, London, UK

## Abstract

**Background:**

*Helicobacter pylori *is the causative agent for gastritis, and peptic and duodenal ulcers. The bacterium displays 5-6 polar sheathed flagella that are essential for colonisation and persistence in the gastric mucosa. The biochemistry and genetics of flagellar biogenesis in *H. pylori *has not been fully elucidated. Bioinformatics analysis suggested that the gene HP0256, annotated as hypothetical, was a FliJ homologue. In *Salmonella*, FliJ is a chaperone escort protein for FlgN and FliT, two proteins that themselves display chaperone activity for components of the hook, the rod and the filament.

**Results:**

Ablation of the HP0256 gene in *H. pylori *significantly reduced motility. However, flagellin and hook protein synthesis was not affected in the HP0256 mutant. Transmission electron transmission microscopy revealed that the HP0256 mutant cells displayed a normal flagellum configuration, suggesting that HP0256 was not essential for assembly and polar localisation of the flagella in the cell. Interestingly, whole genome microarrays of an HP0256 mutant revealed transcriptional changes in a number of genes associated with the flagellar regulon and the cell envelope, such as outer membrane proteins and adhesins. Consistent with the array data, lack of the HP0256 gene significantly reduced adhesion and the inflammatory response in host cells.

**Conclusions:**

We conclude that HP0256 is not a functional counterpart of FliJ in *H. pylori*. However, it is required for full motility and it is involved, possibly indirectly, in expression of outer membrane proteins and adhesins involved in pathogenesis and adhesion.

## Background

*Helicobacter pylori *is a Gram-negative bacterium, colonising the human gastric mucosa. It is responsible for diverse duodenal- and stomach-related disorders, such as ulcers, B cell MALT lymphoma and gastric adenocarcinoma [1-4]. Motility of this bacterium is accomplished by polar sheathed flagella and has been shown to be essential for colonisation, based on animal infection studies [5,6]. Flagella are also involved in adhesion and induction of inflammatory response in the host [7]. Since motility is a virulence-related trait, improving our understanding of flagellum biogenesis in *H. pylori *might help develop intervention strategies or therapeutics.

*H. pylori *flagellar gene transcription is tightly controlled by three RNA polymerase sigma factors σ^80^, σ^54 ^and σ^28 ^[8,9]. σ^80 ^controls the transcription of class I genes (early flagellar genes). σ^54 ^(RpoN) is responsible for the transcription of class II genes (middle flagellar genes). RpoN transcriptional activity is dependent on additional regulators, such the FlgR/FlgS system and the chaperone HP0958 [10-12]. Class III genes (late flagellar genes) are under the control of σ^28 ^(FliA) and the anti-sigma factor FlgM [13,14].

The flagellar export system is recognized as a version of type III secretion systems [15], and during flagellar assembly, it delivers structural components from the cytoplasm to the cell surface and growing organelle. This mechanism is dependent upon export chaperones that protect and deliver structural subunits to the membrane-associated export ATPase, FliI. In *Salmonella*, several flagellar chaperones have been identified. FlgN has chaperone activity for the hook proteins FlgL and FlgK. The chaperone FliT is dedicated to the capping protein FliD, and FliS to the flagellin FliC [16-18]. The ablation of genes encoding FlgN, FliT and FliS impairs the stability and the secretion of their dedicated substrates FlgK, FlgL, FliC and FliD [16,19]. Flagellar biogenesis has been extensively investigated in *Salmonella *and *E. coli *[15,20,21]. Annotation of two *H. pylori *genomes identified homologues of most flagellar genes of the *Salmonella*/*E. coli *paradigm [22-25]. However, some flagellar homologues have not been found in *H. pylori*, presumably due to low sequence identity. Previous bioinformatics searches, targeting only functional domains, were successfully performed to identify the anti-sigma factor FlgM [13,14], and FliK was also identified by a bioinformatic approach [26].

In an effort to identify novel flagellar genes in sequenced *H. pylori *genomes, bioinformatic analysis focusing on identification of specific and conserved domains of flagellar genes was performed. In *Salmonella*, FliJ is a 17 kDa protein with a relative abundance of charged residues. Fraser and colleagues showed that FliJ in *Salmonella *interacts with FliH (the presumptive inhibitor of the FliI ATPase) and FlhA (a flagellar biosynthesis protein) [27]. FliJ was initially thought to display chaperone activity [28]. However, a recent study clearly indicated that FliJ is not a export chaperone for subunits of the hook and the filament [29]. FliJ binds to export chaperones FlgN and FliT and is involved in an escort mechanism, whereby FliJ promotes cycling of the export chaperones FlgN and FliT. A FliJ homologue was not found in the initial annotation of two *H. pylori *genomes, nor incidentally were homologues for FlgN or FliT [22,23,25]. Although searches based on the full-length sequence of FliJ did not identify any *H. pylori *homologues, a search using only the essential FliJ domain (N-terminal coiled-coil domain) did reveal a potential homologue (P. W. O'Toole, unpublished). This analysis identified HP0256, encoding a hypothetical protein with unknown function and a predicted coiled coil domain.

In the present study, we phenotypically characterized a mutant lacking the HP0256 gene product and investigated the function of HP0256 in the flagellar regulon using global transcript analysis. The data suggest a novel role for HP0256 in motility but not flagellum assembly, and involvement in production of cell surface proteins.

## Results

### Bioinformatic analysis of HP0256

**Figure 1:**
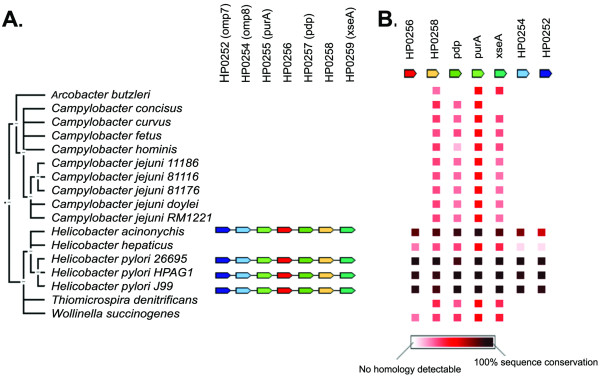
**Gene synteny of HP0256 is conserved in *Helicobacter *genus (Panel A)**. Most HP0256 homologs were found in epsilonproteobacteria (Panel B). Schematics were generated using STRING from EMBL (string.embl.de/).

**Figure 2:**
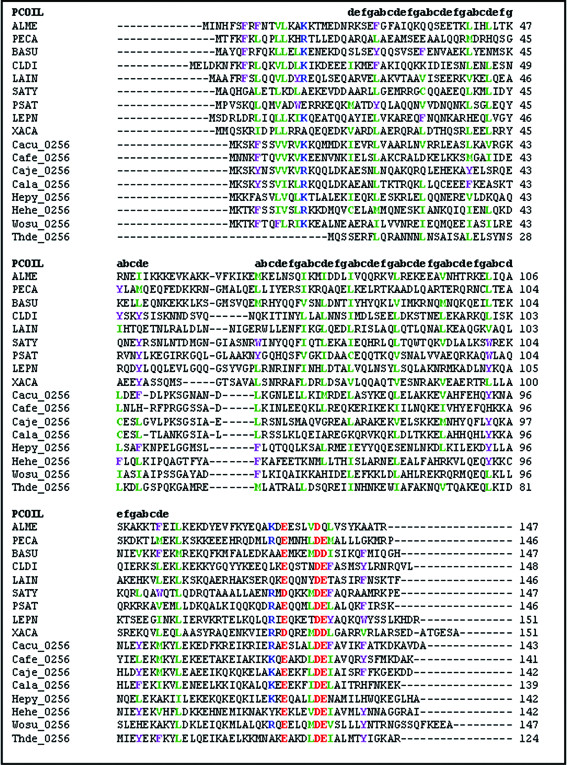
**Multiple sequence alignments of the *H. pylori *HP0256 sequences and orthologues**. The alignment was created using the GENEDOC programme. Residues in colour are conserved in sequences. Sequence regions labelled abcdefg have a high likelihood of forming coiled-coil domains. ALME, gene encoding the flagellar export protein FliJ of *Alkaliphilus metalliredigens*; PECA, gene encoding a putative flagellar biosynthesis chaperone FliJ of *Pelobacter carbinolicus*; BASU, gene encoding a flagellar biosynthesis chaperone of *Bacillus subtilis*; CLDI, gene encoding a flagellar protein of *Clostridium difficile*; LAIN, gene encoding a flagellar biosynthesis chaperone of *Lawsonia intracellularis*; SATY, gene encoding a flagellar biosynthesis chaperone of *Salmonella enterica *subsp. enterica serovar; PSAT, gene encoding the flagellar export protein FliJ of *Pseudoalteromonas atlantica*; LEPN, gene encoding the flagellar protein FliJ of *Legionella pneumophila*; XACA, gene encoding the protein FliJ of *Xanthomonas axonopodis*; Cacu_0256, gene encoding a PP-loop family protein of *Campylobacter curvus*; Cafe_0256, gene encoding an hypothetical protein of *Campylobacter fetus*; Caje_0256, gene encoding an hypothetical protein of *Campylobacter jejuni*; Cala_0256, gene encoding an hypothetical protein of *Campylobacter lari*; Hepy_0256, gene encoding an hypothetical protein of *Campylobacter jejuni*; Hehe_0256, gene encoding an hypothetical protein of *Helicobacter hepaticus*; Wosu_0256, gene encoding an hypothetical protein of *Wolinella succinogenes*; Thde_0256, gene encoding a conserved hypothetical protein of *Thiomicrospira denitrificans*.

PSI-BLAST searches using the full length FliJ sequence from *Salmonella *did not identify any homologues in *H. pylori*. However, using only the FliJ N-terminal coiled-coil domain as a search query, HP0256 was identified as a potential FliJ homologue. The annotation of this *H. pylori *ORF indicates a hypothetical protein with an unknown function. Both FliJ and HP0256 proteins have a similar size (*Salmonella *FliJ, 147 amino-acids; HP0256, 142 amino-acids) and have a high likelihood of forming N-terminal coiled-coils. They share 17% identity and 44% similarity. In contrast, FliJ from *Salmonella *and *E. coli *are 88% identical and 96% similar. Further searches identified potential HP0256 homologues in more related species (Figure 1). An alignment of these is shown in Figure 2. HP0256 is 22% identical and 51% similar to WS2055 of *Wolinella succinogenes*, 28% identical and 51% similar to ZP_01374471 of *Campylobacter concisus *and 23% identical and 65% similar to CJ1497c of *Campylobacter jejuni*.

For each of the FliJ and HP0256 sequence groups, both Paircoil2 and PCOILS were run (for PCOILS, the multiple sequence alignment used to generate Figure 2 was used) [30]. For Paircoil2, approximately 10 FliJ annotated sequences, ranging from 35 to 15% overall identity, were used. Each sequence gave essentially the same profile, and the program output yielded the same region (plus or minus 5 residues on average) with the same heptad register. Hence the predicted coiled coil domains were internally consistent for the FliJ family and the HP0256 family. In addition, the predicted coiled coil domains matched between families [31].

The gene for the *Salmonella *FliJ protein is flanked by those of the FliI ATPase and the hook length control protein FliK, as part of the FliE operon. Flagellar genes in *H. pylori *are not contained in such large operons, but are scattered throughout the genome [23,32]. HP0256 is flanked by an adenylosuccinate synthetase gene (*purA*/HP0255) as well as two outer membrane protein genes (*omp7*/HP0252 and *omp8*/HP0254), and three hypothetical genes, one of which encodes a predicted secreted protein (HP0257) and the other a predicted integral membrane protein (HP0258). When comparing HP0256 with homologues from related species, it did not appear that any one domain of the protein was more or less conserved (Figure 2). This agrees with previous studies of FliJ data suggesting that the entire protein is necessary for function [28]. As this bioinformatic analysis suggested HP0256 could be a FliJ homologue, we generated a HP0256 mutant by inserting a chloramphenicol resistance marker into the gene by allelic exchange as described in Methods. Growth rates and plate morphology of the HP0256 mutant were indistinguishable from the wild-type (data not shown).

### Ablation of the HP0256 gene reduces motility

**Figure 3:**
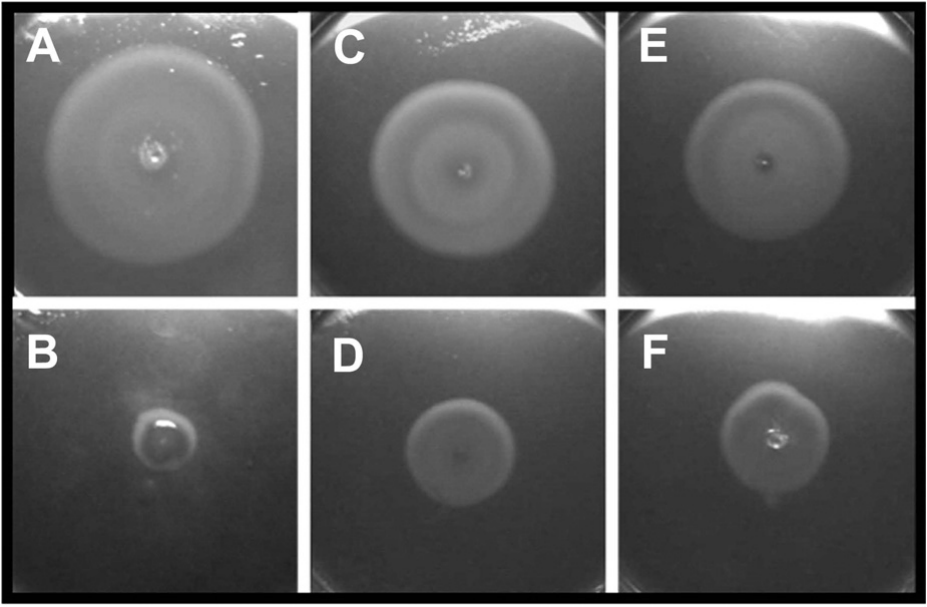
**The ablation of the HP0256 gene impairs motility in *H. pylori *that may be restored by complementation, when *hp0256 *is put under the control of the promoter of *flaA***. Motility plate assay were performed four times. A. CCUG17874 wild-type strain; B. CCUG17874-*hp0256*KO; C. P79 wild-type strain; D. P79-*hp0256*KO; E. P79-*hp0256*KO complemented with pIR0601; F. P79-*hp0256*KO with empty vector (control).

Motility plate assay indicated that the HP0256 mutant was significantly less motile than the wild-type (Figure 3). A similar phenotype was consistently observed in two *H. pylori *wild-type strains and their derivative HP0256 mutants (Figure 3), indicating that the reduced motility was not a strain-specific effect. However, the mutants retained some motility. In *Salmonella*, lack of FliJ abolishes motility [27], suggesting that HP0256 may not be a FliJ homologue as initially hypothesized. Complementation of a *Salmonella *FliJ mutant was attempted by introduction of the HP0256 gene expressed from an *E. coli *vector promoter. Motility plate assay indicated that motility was not restored in the *Salmonella fliJ *mutant, indicating that HP0256 was unlikely to be a functional FliJ homologue in *Helicobacter pylori *(data not shown). We complemented the P79-derivative HP0256 mutant, by expressing the HP0256 gene, integrated into the chromosome, under the control of the *flaA *promoter (Figure 3). Restoration of motility in the complemented mutant confirmed that the partial loss of motility in the mutant was due only to the lack of the HP0256 gene product.

### An HP0256 mutant produces normal levels of flagellin and hook proteins

**Figure 4:**
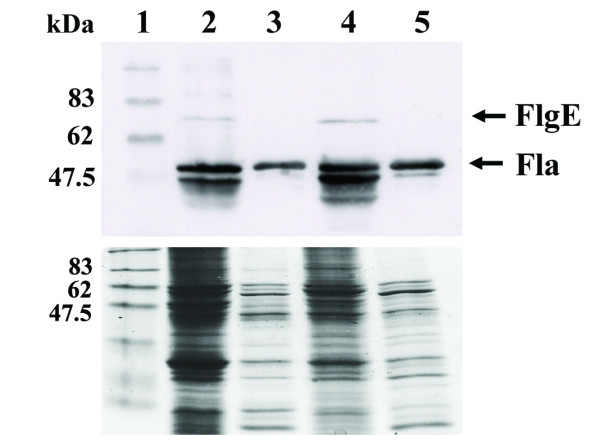
**Mutation of HP0256 does not affect flagellin and hook protein production**. Flagellin and hook protein levels in the HP0256-KO mutant and the wild-type were analyzed by SDS-PAGE and immunoblotting. Two independent immunoblottings were performed. Panel A, Coomassie blue staining protein gel, Panel B, immunobloting, Lane 1, Protein marker; lane 2, CCUG17874 cytoplasmic fraction; lane 3, CCUG17874 cell envelope fraction; lane 4, cytoplasmic fraction of CCUG17874 derivative HP0256-KO mutant and lane 5, cell envelope fraction of CCUG17874 derivative HP0256-KO mutant.

The partial loss of motility in the HP0256 mutant might have been due to altered production levels of major flagellar components. *H. pylori *flagellum filaments are made of two proteins, a major flagellin FlaA and a minor flagellin FlaB. The hook consists of FlgE protein. We investigated flagellin and hook protein production in an HP0256 mutant using immunoblotting analysis with anti-*H. pylori *flagellin antiserum [33]. The antiserum used for immunoblotting is reactive with both flagellins and the hook protein. Minamino *et al*. had previously described a *Salmonella *FliJ defective mutant which had less flagella than wild-type cells [28]. In contrast with a *Salmonella *FliJ mutant, we could not observe any significant difference in the amount of flagellin protein in the cytoplasm or envelope protein fractions of the HP0256 mutant compared to corresponding fractions from wild-type cells (Figure 4). The normal production of FlgE protein compared to the *flgE *up-regulation may be due to a post-transcriptional regulation. Interestingly, it appeared that there were more degradation products in the HP0256 mutant samples compared to the wild-type, and this was consistently observed in technical and biological replicates of the immunoblotting analyses we performed (not shown).

### An HP0256 mutant displays a normal flagellum configuration

**Figure 5:**
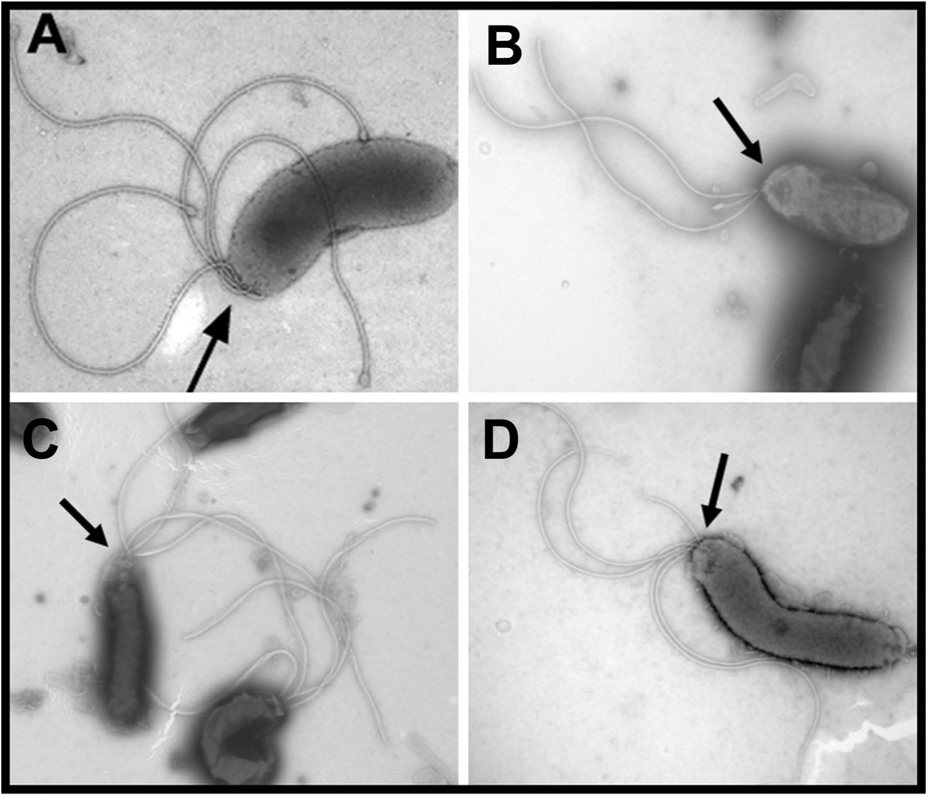
**An HP0256 mutant has a normally assembled flagellum filament**. The arrows indicate the localisation of the flagella in the cell. The transmission electron microscopy was performed on 50 cells for each strain. Panel A, CCUG17874 wild-type; panel B, P79 wild-type; panel C, CCUG17874-*hp0256*KO and panel D, P79-*hp0256*KO.

Another plausible explanation for the reduced motility in the HP0256 mutant would be the presence of flagella with an aberrant morphology. We therefore performed transmission electron microscopy to investigate the flagellum configuration in wild-type and mutant cells. Wild-type *H. pylori *CCUG17874 and P79 cells harboured 2-3 polar flagella (Figure 5). In the HP0256 mutant cells, the number and localization of flagella were similar to the wild-type cells (Figure 5). Flagella of the mutant cells had the same length as those on wild-type cells. They were sheathed and had normal flagellar hooks.

### Transcriptional analysis of an HP0256 mutant

**Figure 6:**
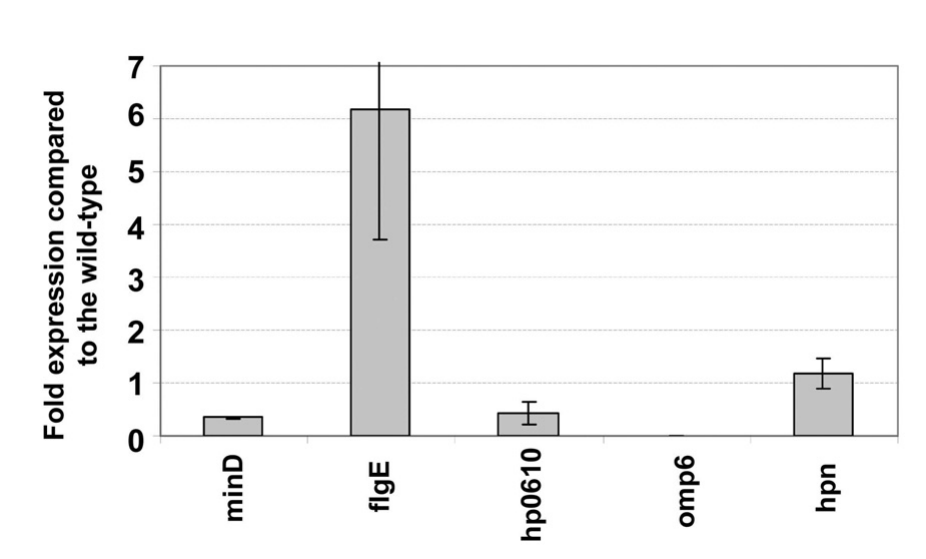
**Confirmation of transcriptional changes in selected flagellar genes in the HP0256 mutant using qRT-PCR**. Fold changes and standard deviations were calculated using the *era *transcript abundance as reference. qRT-PCRs were performed on at least two biological replicates.

The flagellar circuitry in *H. pylori *consists of three sigma factors and other regulators, such as the anti-σ^28 ^factor FlgM and the FlgR/FlgS activation system [8,34]. The lack of one regulatory player can deregulate the whole flagellar biosynthetic cascade and alter motility in *H. pylori*. Since ablation of the HP0256 gene reduced motility, we investigated the effect of HP0256 mutation upon the expression of the flagellar regulon using global transcript analysis. Array analysis was performed in quadruplicate, including a dye-swap. Five genes were selected to confirm the reliability of our microarray data by qRT-PCR. Transcriptional level of *hpn *was unchanged in the HP0256 mutant and was therefore used a control for qRT-PCR. The fold changes thus established were in good agreement with the array data (Figure 6). The difference observed in fold-changes of *flgE *transcription between array data and qRT-PCR is due to the microarray analysis method used for the study. This method tends to attenuate the dispersion of the fold-changes compared to the overall signal on the slide.

A total of forty six genes had altered expression levels in the HP0256 mutant. Nineteen genes were significantly up-regulated and twenty seven genes down-regulated in the HP0256 mutant compared to the wild-type strain (Table 1). Data for some biologically relevant genes, below the two-fold cut-off, are also included in Table 1. Among the differentially expressed genes, seventeen encode proteins associated with the membrane.

**Table 1:** Gene list of significantly up- and down-regulated genes in the HP0256 mutant based on the array experiment.

**TIGR orf no**.	Putative gene product (gene)	Expression ratio	p-value
**Down-regulated genes:**			
Hp26695-0092	type II restriction enzyme M protein (*hsdM*)	0.15	0.02
HpJ99-1132	dimethyladenosine transferase	0.17	0.00
Hp26695-0093	**alpha-2-fucosyltransferase**	0.22	0.01
Hp26695-0229	**outer membrane protein (omp6) (*hopA*)**	0.24	0.01
Hp26695-0492	**flagellar sheath associated protein (*hpaA3*)**	0.26	0.00
Hp26695-1210	serine acetyltransferase (*cysE*)	0.26	0.00
Hp26695-1587	conserved hypothetical protein	0.27	0.00
Hp26695-1208	ulcer associated adenine specific DNA methyltransferase	0.27	0.00
Hp26695-0610	**toxin-like outer membrane protein**	0.32	0.03
Hp26695-1207	hypothetical protein	0.34	0.01
HpJ99-0055	putative	0.35	0.03
Hp26695-1211	hypothetical protein	0.37	0.00
Hp26695-0430	hypothetical protein	0.40	0.04
Hp26695-1492	conserved hypothetical nifU-like protein	0.41	0.01
Hp26695-0805	**lipooligosaccharide 5G8 epitope biosynthesis-associated protein**	0.42	0.02
Hp26695-1203a	Preprotein translocase subunit SecE	0.43	0.01
Hp26695-1219	hypothetical protein	0.43	0.04
Hp26695-0711	hypothetical protein	0.45	0.01
Hp26695-1180	pyrimidine nucleoside transport protein (*nupC*)	0.46	0.03
Hp26695-0502	hypothetical protein	0.46	0.03
Hp26695-1589	conserved hypothetical protein	0.47	0.01
Hp26695-0094	**alpha-2-fucosyltransferase**	0.49	0.02
Hp26695-1334	hypothetical protein	0.49	0.01
Hp26695-0415	**conserved hypothetical integral membrane protein**	0.49	0.01
Hp26695-0340	hypothetical protein	0.49	0.00
Hp26695-0798	molybdenum cofactor biosynthesis protein C (*moaC*)	0.49	0.03
Hp26695-0892	conserved hypothetical protein	0.50	0.03
Hp26695-0331	**cell division inhibitor (*minD*)**	0.59	0.04
			
**Up-regulated genes:**			
Hp26695-0115	**flagellin B (*flaB*)**	1.91	0.03
Hp26695-0979	**cell divison protein (*ftsZ*)**	1.92	0.00
Hp26695-1469	**outer membrane protein (*omp31*) (*hopV*)**	1.96	0.00
Hp26695-1243	**outer membrane protein (*omp28*) (*babA*)**	1.96	0.00
Hp26695-0386	hypothetical protein	2.01	0.00
Hp26695-0831	conserved hypothetical ATP binding protein	2.04	0.01
Hp26695-0952	**conserved hypothetical integral membrane protein**	2.05	0.00
Hp26695-0311	hypothetical protein	2.16	0.00
Hp26695-0720	hypothetical protein	2.16	0.02
Hp26695-0943	D-amino acid dehydrogenase (dadA)	2.18	0.01
Hp26695-0896	**outer membrane protein (*omp19*) (*babB*)**	2.18	0.00
Hp26695-0590	ferredoxin oxidoreductase, beta subunit	2.23	0.01
Hp26695-0589	ferredoxin oxidoreductase, alpha subunit	2.27	0.01
Hp26695-1340	**biopolymer transport protein (*exbD*)**	2.30	0.00
Hp26695-1339	**biopolymer transport protein (*exbB*)**	2.36	0.00
Hp26695-0747	conserved hypothetical protein	2.44	0.03
Hp26695-0310	conserved hypothetical protein	2.48	0.00
Hp26695-1322	hypothetical protein	2.57	0.03
Hp26695-1076	**hypothetical protein**	2.59	0.00
Hp26695-1524	hypothetical protein	2.68	0.05
Hp26695-0721	hypothetical protein	2.99	0.00
Hp26695-0744	pseudogene	3.08	0.00
Hp26695-0719	hypothetical protein	3.34	0.01
Hp26695-0954	oxygen-insensitive NAD(P)H nitroreductase	3.53	0.00

The fold-change and the p-value are indicated. Bold fonts were used to highlight genes considered biologically relevant for the present study (surface-or motility-related genes). Full array datasets are in public databases as described in Methods.

Interestingly, four genes encoding proteins of the Hop outer membrane family were identified as differentially expressed in the HP0256 mutant by microarray analysis (*hopA*/HP0229, *hopV*/HP1469, *babA*/HP1423 and *babB*/HP0896). *hopA *was four fold down-regulated, whereas the other three Hop genes were up-regulated. HP1339 and HP1340, encoding respectively the biopolymer transport proteins ExbB and ExbD, were up-regulated in the HP0256 mutant. ExbB and ExbD in *E. coli *interact with the TonB-dependent energy transduction complex [35]. In *E. coli*, TonB is involved in the transduction of energy between the cytoplasmic membrane and the outer membrane [36]. Five genes involved in lipopolysaccharide (LPS) production were differentially expressed: HP0093 (alpha-(1,2)-fucosyltransferase), HP0094 (alpha-(1,2)-fucosyltransferase), HP0805 (lipooligosaccharide biosynthesis-associated protein) and HP0310 (contains a polysaccharide deacetylase Pfam domain).

We identified a number of flagellar genes with altered expression levels (Tables 1 and 2). Three RpoN-dependent genes were significantly up-regulated in the HP0256 mutant based on the microarray data and the qRT-PCR investigations, *i.e*. HP0115/*flaB *(encoding the minor flagellin FlaB), HP0870/*flgE *(encoding the hook protein FlgE) and HP1076 (encoding a hypothetical protein). Another RpoN-dependent gene HP1155/*murG *(transferase, peptidoglycan synthesis) was 1.955 fold up-regulated with a *p*-value of 0.034. However, RpoN and its associated regulators FlgR, HP0244 and HP0958 were transcribed at wild-type levels. As shown in Table 2, HP0492/*hpaA3 *(flagellar sheath associated protein) was significantly down-regulated. This gene is known to be essential for flagellar biogenesis, but its transcriptional regulation remains unclear. It has not yet been assigned to any flagellar gene class [8]. In the intermediate class, HP0367 (encoding a hypothetical protein) was 1.8 fold up-regulated with a p-value of 0.008. In class I genes, we did not observe significant changes. A slight down-regulation of genes encoding components of the secretion apparatus and the basal body, such as FliI, FliQ, FliB, FlgG, was noted without reaching the fold-change cut-off for significance. The *fliN *gene encoding a component of the switch was up-regulated (1.758 fold) with a p-value of 0.042.

**Table 2:** Differentially expressed flagellar genes in the HP0256 mutant.

Proposed Class	**TIGR orf no**.	Putative gene product (gene)	Expression ratio	p-value
Class I	HP0019	chemotaxis protein (*cheV*)	**1.221**	**0.026**
	HP0082	methyl-accepting chemotaxis transducer (*tlpC*)	0.945	0.378
	HP0099	methyl-accepting chemotaxis protein (*tlpA*)	1.401**	0.112
	HP0103	methyl-accepting chemotaxis protein (*tlpB*)	1.403**	**0.05**
	HP0173	flagellar biosynthetic protein (*fliR*)	1.000	0.997
	HP0244	signal-transducing protein, histidine kinase (*atoS*)	1.221	0.651
	HP0246	flagellar basal-body P-ring protein (*flgI*)	-	-
	HP0325	flagellar basal-body L-ring protein (*flgH*)	1.113	0.050
	HP0326	CMP-N-acetylneuraminic acid synthetase (*neuA*)	0.904	0.219
	HP0327	flagellar protein G (*flaG*)	0.749	0.238
	HP0351	basal body M-ring protein (*fliF*)	0.889	0.508
	HP0352	flagellar motor switch protein (*fliG*)	1.158	0.176
	HP0391	purine-binding chemotaxis protein (*cheW*)	1.668**	**0.004**
	HP0392	histidine kinase (*cheA*)	1.202	0.113
	HP0393	chemotaxis protein (*cheV*)	1.176	0.194
	HP0584	flagellar motor switch protein (*fliN*)	1.758**	**0.042**
	HP0599	hemolysin secretion protein precursor (*hylB*)	1.201	0.366
	HP0616	chemotaxis protein (*cheV*)	1.159**	0.162
	HP0684	flagellar biosynthesis protein (*fliP*)	0.510	0.058
	HP0685	flagellar biosynthetic protein (*fliP*)	0.493	0.066
	HP0703	response regulator	0.715	0.158
	HP0714	RNA polymerase sigma-54 factor (*rpoN*)	1.104	0.699
	HP0770	flagellar biosynthetic protein (*flhB*)	0.621	0.162
	HP0815	flagellar motor rotation protein (*motA*)	0.917	0.538
	HP0816	flagellar motor rotation protein (*motB*)	0.651	0.231
	HP0840	*flaA1 protein*	1.296	0.184
	HP1041	flagellar biosynthesis protein (*flhA*)	0.988	0.921
	HP1067	chemotaxis protein (*cheY*)	0.958	0.905
	HP1092	flagellar basal-body rod protein (*flgG*)	1.142	0.140
	HP1286	conserved hypothetical secreted protein (*fliZ*)	1.305	0.544
	HP1419	flagellar biosynthetic protein (*fliQ*)	**0.636**	**0.036**
	HP1420	flagellar export protein ATP synthase (*fliI*)	**0.687**	**0.012**
	HP1462	secreted protein involved in flagellar motility	**1.306**	**0.003**
	HP1575	homolog of FlhB protein (*flhB2*)	1.445	0.239
	HP1585	flagellar basal-body rod protein (*flgG*)	**0.590**	**0.019**

Class II	HP0114	hypothetical protein	1.230	0.357
	HP0115	flagellin B (*flaB*)	**1.906**	**0.032**
	HP0295	flagellin B homolog (*fla*)	1.734	0.179
	HP0869	hydrogenase expression/formation protein (*hypA*)	1.307	0.109
	HP0870	flagellar hook (*flgE*)	1.892*	0.067
	HP0906	hook length control regulator (*fliK*)	1.13**	0.230
	HP1076	hypothetical protein	**2.595**	**0.001**
	HP1119	flagellar hook-associated protein 1 (HAP1) (*flgK*)	1.300	0.224
	HP1120	hypothetical protein	1.199	0.390
	HP1154	hypothetical protein (operon with *murG*)	1.514	0.055
	HP1155	transferase, peptidoglycan synthesis (*murG*)	**1.955**	**0.034**
	HP1233	putative flagellar muramidase (*flgJ*)	1.400	0.144

Class III	HP0472	outer membrane protein (*omp11*)	**1.649**	**0.009**
	HP0601	flagellin A (*flaA*)	1.487	0.229
	HP1051	hypothetical protein	1.098	0.501
	HP1052	UDP-3-0-acyl N-acetylglucosamine deacetylase (*envA*)	1.648	0.054

Intermediate	HP0165	hypothetical protein	1.226	0.515
	HP0166	response regulator (*ompR*)	1.596	0.057
	HP0366	spore coat polysaccharide biosynthesis protein C	0.860	0.419
	HP0367	hypothetical protein	**1.853**	**0.008**
	HP0488	hypothetical protein	0.711**	**0.031**
	HP0907	hook assembly protein, flagella (*flgD*)	1.271	0.214
	HP0908	flagellar hook (*flgE*)	1.175	0.119
	HP1028	hypothetical protein	0.852	0.286
	HP1029	hypothetical protein	**0.799**	**0.019**
	HP1030	fliY protein (*fliY*)	0.860**	0.308
	HP1031	flagellar motor switch protein (*fliM*)	0.835	0.054
	HP1032	alternative transcription initiation factor, sigma28 (*fliA*)	0.923	0.371
	HP1033	hypothetical protein	0.896	0.467
	HP1034	ATP-binding protein (*ylxH*)	0.87**	0.352
	HP1035	flagellar biosynthesis protein (*flhF*)	0.921	0.187
	HP1122	anti-sigma 28 factor (*flgM*)	0.867	0.310
	HP1440	hypothetical protein	0.627	0.026
	HP1557	flagellar basal-body protein (*fliE*)	0.652	0.091
	HP1558	flagellar basal-body rod protein (*flgC*) (proximal rod protein)	0.899	0.480
	HP1559	flagellar basal-body rod protein (*flgB*) (proximal rod protein)	1.305	0.194
	HP0751	(*flaG2*)	1.203	0.350
	HP0752	flagellar cap protein (*fliD*)	1.003	0.986
	HP0753	flagellar chaperone (*fliS*)	0.981	0.825
	HP0754	flagellar chaperone (*fliT*)	1.09**	0.400

Not assigned	HP0410	flagellar sheath associated protein (*hpaA2*)	**0.664**	**0.038**
	HP0492	flagellar sheath associated protein (*hpaA3*)	**0.256**	**0.000**
	HP0797	flagellar sheath associated protein (*hpaA*)	0.801	0.170

Full array datasets are in public databases as described in Methods. Fold-changes and p-values were calculated based on 4 independent biological replicates as described in Methods. Open reading frames and gene annotations were based on the TIGR database [23]. The genes were classified in different flagellar classes, as previously proposed [8]. Confirmatory analysis by qRT-PCR was performed for genes with *. Values for genes with ** were lost during the initial array data analysis and subsequently recovered using 3 independent replicates. For technical reasons, some array spots could not be analyzed in individual arrays.

Two genes involved in the cell division process were affected in the HP0256 mutant. HP0331/*minD*, coding for a protein involved in the correct localisation of the cell division site [37], was 1.7 fold down-regulated in the HP0256 mutant compared to the wild-type (confirmed by qRT-PCR investigation). In *E. coli*, MinD (in synergy with MinC) inhibits the cell division protein FtsZ, that forms the FtsZ or Z ring at the septum [38,39]. Interestingly, *ftsZ *was 1.9 fold up-regulated in the HP0256 mutant (Table 1).

### Adhesion and pro-inflammatory properties of an HP0256 mutant

**Figure 7:**
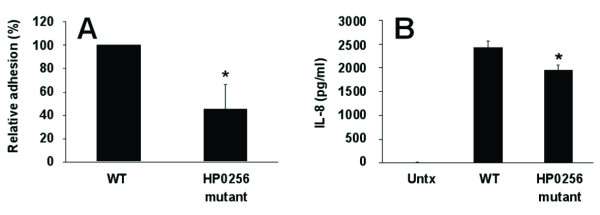
**The HP0256 mutant has lower adhesion ability compared to the wild-type and significantly induces a weaker IL-8 secretion in AGS cells**. Panel A shows that the HP0256 mutant adheres significantly less to the AGS host cells compared to the wild-type. Panel B shows that the HP0256 mutant induces a lower IL-8 secretion of AGS cells compared to the wild-type cells. (*) indicates results with a *p*-value of less than 0.05.

The microarray data indicated altered expression of a number of genes encoding proteins associated with the cell envelope in the HP0256 mutant. The genes encoding the well-characterized adhesins BabA and BabB which bind to fucosylated Lewis antigens on human gastric cells were up-regulated in the HP0256 mutant. To investigate a potential role of HP0256 in pathogenesis and adhesion, we measured adhesion of HP0256 mutant cells to gastric epithelial cells, and also interleukin-8 (IL-8) secretion by gastric epithelial cells using an *in vitro *infection model. Adhesion of the HP0256 mutant to AGS cells was significantly reduced to 45% of that of the wild-type (p < 0.05) (Figure 7). Supernatants from that assay were also used to quantify IL-8 production by AGS cells. CCUG17874 induced an average of 2434 pg/ml of IL-8 from AGS cells compared to 1944 pg/ml by the HP0256 mutant (Figure 7). This is a statistically significant decrease of 20% (p < 0.02).

## Discussion

A focused bioinformatics analysis based on the functional domain of FliJ (N-terminal coiled-coil domain) suggested that HP0256 was a potential FliJ homologue in *H. pylori*. HP0256 encodes a hypothetical protein in *H. pylori *and shares common properties with FliJ, such as a similar size and a predicted N-terminal coiled coil. However, in comparison with the complete loss of motility reported in a *Salmonella *FliJ mutant [27], *H. pylori *HP0256 mutants retained some motility based on a motility plate assay. Complementation of a *Salmonella *FliJ mutant was attempted by introduction of the HP0256 gene product expressed under the control of an *E. coli *promoter, but this did not restore motility in the transformed *Salmonella *FliJ mutant (data not shown). Immunoblotting analysis revealed no significant differences in flagellin and hook protein synthesis between the wild-type and the HP0256 mutant. The partial loss of motility in the HP0256 mutant was therefore not due to impairment in filament and hook protein production. The increased degradation rate of flagellar proteins observed in the HP0256 mutant samples compared to the wild-type suggested a possible chaperone activity of HP0256. However, the apparently normal flagellum assembly and localisation at the pole in the HP0256 mutant cells suggested that HP0256 was not actually essential for flagellum protein stabilization or export apparatus positioning. In the HP0256 mutant, the significant reduction in motility still remained unclear. Quantitative data, *e.g*. average time and lengths of swimming runs, to characterize the motility phenotype of the HP0256 mutant would allow us to further comprehend the effect of HP0256 on *Helicobacter pylori *motility. Although this was not mechanistically wholly elucidated, the potential players in this phenotype were identified by array analysis.

Global transcript analysis indicated that HP0256 interferes with the transcription of flagellar genes belonging to the RpoN regulon. Four RpoN-dependent genes were up-regulated in the HP0256 mutant, although transcription of RpoN and its associated regulators FlgR, HP0244 and HP0958 were at wild-type level. The different transcriptional profiles among RpoN-dependent genes suggested that some key RpoN-dependent genes may be under additional regulatory checkpoints, likely HP0256-dependent. However, we do not have data to explain the mechanistic links involved in this regulation. Among class II genes, the only known flagellar regulator HP0906/FliK controls the hook length and is involved in the hook-filament transition. HP0906 was transcribed at wild-type level, in agreement with the normal flagellar morphology in HP0256 mutants (i.e. absence of polyhooks). The up-regulation of four RpoN-dependent genes in the HP0256 mutant did not grossly interfere with flagellar assembly as demonstrated by transmission electron microscopy (normal flagellum configuration in HP0256 mutants). However, a modification of the FlaA/FlaB ratio in flagella significantly affects motility [40] and this may still be responsible for the aberrant functioning of the flagellar organelle seen here.

Interestingly, HP0256 mutant cells were not predominantly swimming but tumbling, based on light microscopy observations. This abnormal motility behaviour, which may explain the reduced motility in the HP0256 mutant, underlined a probable disruption of the switch mechanism between swimming and tumbling. FliG, FliM and FliN are involved in switching between clockwise and counter-clockwise rotation of the motor and thus, of the filament [41,42]. It is noteworthy that FliN was upregulated. Other components involved in the switch, FliM and FliG, were normally expressed. The FliM and FliN proteins assemble to form a ring, called the C-ring [41]. In *Salmonella*, the FliN protein is involved in the switch process and its interaction with FliH is crucial for the localisation of the FliI-FliH complex in the C-ring [43]. We hypothesize that the 1.758-fold overexpression of FliN may be sufficient to modify the stoichiometry of the switch subunits, disrupting the correct functioning of the switch. The HP0256 mutant cells would then be unable to properly respond to chemotactic environmental stimuli, as illustrated by the abnormal motility observed in the HP0256 mutant. A slight caveat for this hypothesis is that we do not have data to confirm an increase of FliN protein production in the HP0256 mutant.

A number of outer membrane proteins and LPS-related proteins were differentially expressed in the HP0256 mutant. BabA and BabB expression were both up-regulated in the HP0256 mutant. BabA binds to the blood group antigen Lewis b [44]. The sialic acid-specific adhesin HpaA is enriched in the flagellar sheath [45] and was significantly down-regulated in the HP0256 mutant. HpaA has been shown to be antigenic but not involved in the interaction with AGS cells [45]. The modifications of the cell envelope architecture, *i.e*. adhesins, hop proteins, alpha-2-fucosyltransferase, may explain the reduced ability of the HP0256 mutant to adhere to host cells and to induce an inflammatory response, *i.e*. interleukin-8 secretion. The disruption of HP0256 and its effect on cell envelope architecture may modify the lipid profiles and/or membrane fluidity and therefore the function of the methyl-accepting chemotactic proteins. The biological significance of the alteration of expression of *minD *and *ftsZ *in the HP0256 mutant, two genes involved in the cell division process, remains unclear. A correlation with other membrane-associated protein expression, such as outer membrane proteins, cannot be excluded and additional experiments will be required to test this.

## Conclusions

We initially hypothesized that HP0256 was a FliJ homologue in *H. pylori *based on bioinformatic analyses. Our data clearly show that HP0256 has a different function in *H. pylori*, compared to that of FliJ in *Salmonella*. Interestingly, HP0256 is still obviously involved in flagellum activity as its ablation caused a partial loss of motility. Its involvement with expression of some RpoN-dependent genes is noteworthy but did not result in major changes in the mutant phenotype (normal flagellar apparatus configuration). The partial loss of motility must therefore be due to effects upon other flagellar players. Based upon its observed up-regulation in the HP0256 mutant, FliN is a potential candidate responsible for the impaired motility we observed in the HP0256 mutant. Further investigation of FliN production in the HP0256 mutant or overexpression in the wild-type would confirm this hypothesis. The large number of membrane-associated proteins with an altered expression in the HP0256 mutant highlighted another aspect of the mutant phenotype: the alteration of the cell envelope architecture, likely responsible for the weak adhesion to, and the low inflammatory response induced in, host cells. We conclude that HP0256 is required for full motility of *H. pylori*, possibly through its involvement with the switch components, but that it also modulates directly or indirectly the normal expression of membrane proteins essential in pathogenesis.

## Methods

### Bacterial strains, media and growth conditions

Bacterial strains used in this study are listed in Table 3. *H. pylori *strain P79 [46], a streptomycin mutant of the P1 wild-type strain, was generously provided by Dr. R. Haas. *H. pylori *strains were cultured as previously described [26]. Two *H. pylori *mutants lacking the HP0256 gene (one in CCUG17874 and one in P79) were generated as described below in Materials and Methods. Transformants were selected on CBA (Columbia agar base) plates supplemented with 10 μg/ml chloramphenicol (Sigma) and/or 50 μg/ml kanamycin (Sigma). One Shot TOP10 chemically competent *E. coli *cells (Invitrogen, CA, USA) were propagated on Luria-Bertani (LB) agar plates or LB broth at 37°C supplemented with antibiotics: 50 μg/ml kanamycin (Sigma), 100 μg/ml ampicillin (Merck, Germany) and 10 μg/ml chloramphenicol (Sigma).

**Table 3:** Strains and plasmids used in this study.

Strains or plasmids	Relevant characteristics	Reference or source
*Strains*		
		
*H. pylori*		
CCUG17874	wild-type strain	CCUG, Sweden
*hp0256 *KO	CCUG17874 Δ*hp0256*::Cm^r^	This study
P1	wild-type strain	[57]
P79	P1 Str^r^	[58]
P79-*hp0256*KO	P79 Δ*hp0256*::Cm^r^	This study
P79-0256/pIR203K04	P79 Δ*hp0256*::Cm^r ^with pIR203K04 (Kan^r^)	This study
P79-0256/pIR0601	P79 Δ*hp0256*::Cm^r ^with pIR0610 (Kan^r^)	This study
		
*S. typhimurium*		
SJW1103	wild-type strain	[59]
MKM40	SJW1103 *ΔfliJ*	[59]
MKM40-pQE60	SJW1103 *ΔfliJ *with empty pQE-60	This study
MKM40-pQE60-0256	SJW1103 *ΔfliJ *with pQE-60-0256	This study
		
*E. coli*		
One Shot TOP10	F^- ^*mcr*A Δ(*mrr*-*hsd*RMS-*mcr*BC) ф80*lac*Z ΔM15 Δ*lac*X74 *rec*A1 *ara*Δ139 Δ(*ara*-*leu*)7697 *gal*U *gal*K *rps*L (Str^r^) *end*A1 *nup*G	Invitrogen, USA
		
*Plasmids*		
		
pIR203K04	kanamycin resistance cassette (Kan^r^)	[51]
pIR0601	pIR203K04 with *hp0256 *gene under the control of *hp0601 *promoter	This study
	C-term His-tagged expression vector (Amp^r^)	
pQE-60	pQE-60 with *hp0256 *gene	Qiagen, Germany
pQE-60-0256	This study	

Cm, chloramphenicol, Kan, kanamycin; Str, streptomycin

### Bioinformatics

PSI-BLAST was performed using bacterial sequences from the NCBI non-redundant protein databank at NCBI-BLAST. Three to four iterations were run and false-positives were edited from the output. Searching with *Salmonella *or other FliJ sequences did not result in significant hits with any HP0256 homologues. However, using one of the HP0256 homologues for a PSI-BLAST search often resulted in *Bacillus *FliJ sequences appearing just below the inclusion threshold with E-values ranging between 0.08 and 0.52. In addition, the alignments from these BLAST hits were deemed correct as judged by comparison to the multiple alignment presented in Figure 1. For each of the FliJ and HP0256 sequence groups, both Paircoil2 and PCOILS were run (for PCOILS, the multiple sequence alignment used to generate Figure 1 was used) [30].

### Allelic exchange mutagenesis

*Helicobacter *DNA was isolated as previously described [47]. Oligonucleotides were purchased from Eurofins MWG Operon (Germany). Oligonucleotides ML022FP/ML027RP (Table 4) were designed for the amplification of a 216 bp fragment containing the 3' end of HP0255 and the 5' end of HP0256. Oligonucleotides ML028FP/ML023RP (Table 4) were designed for the amplification of a 245 bp fragment at the 5' end of HP0256. ML027RP and ML028FP had overlapping sequences and included a *BglII *restriction site. The two amplicons were joined together by extension overlap PCR and the resulting DNA product was cloned into pUC18 (New England Biolabs, USA) following *BamHI *and *EcoRI *digestion. The resultant plasmid was cut with *BglII *and ligated with the chloramphenicol acetyl transferase (*cat*) gene which had been cut from the plasmid pRY109 [48]. *H. pylori *cells were transformed with 1 μg of this plasmid for double-cross over gene disruption as previously described [26]. Polymerase chain reactions (PCR) were performed using 3 μM of each primer and 0.5 units per reaction of Vent Polymerase (New England Biolabs).

**Table 4:** Oligonucleotide sequences used in this study.

Primer	Sequence (5'-3')	Gene	Comments
flgE-F	GGCTAACGAGCGTGGATAAG	*flgE*	FP of *flgE*
flgE-R	GAGCGAGCGCTAAAGTCCTA	*flgE*	RP of *flgE*
era-F	AAGGCTAATGCGACCAGAAA	*hp0517*	FP of *era*
era-R	GGAGCCCTGGTGTGTCTAAA	*hp0517*	RP of *era*
ML022FP	CGGGATCCCGGGGCGAAAGATTGGAGATTT	*hp0256*	Allelic exchange mutagenesis
ML027RP	CCATCGTAGATCTGGGCTGC AGCGAATTTTTTCATAGAAAAATCG	*hp0256*	Allelic exchange mutagenesis
ML028FP	GCAGCCCAGATCTACGATGGGCAATTAAAAAGCGCTCTAAGAAT	*hp0256*	Allelic exchange mutagenesis
ML023RP	CGGAATTCCGTTACGCATGCAAGCCCTC	*hp0256*	Allelic exchange mutagenesis
HP0256-F2	TATAACAAGGAGTTACAACAATGAAAAAATTCGCTTCTGTG	*hp0256*	FP of *hp0256*
HP0256-R	GCGCGCATCGATTTACGCATGCAAGCCCTCTT	*hp0256*	RP of *hp0256*
FLA-F2	GCGCGCGGATCCCATGCTCCTTTAAATTTTGC	*flaA*	FP of *flaA *promoter
FLA-R	TGTTGTAACTCCTTGTTATA	*flaA*	RP of *flaA *promoter
minD-F	TAATTTAGCGATCGGCTTGG	*minD*	FP of *minD*
minF-R	TCCATCACATCCACCACATC	*minD*	RP of *minD*
hp0610-F	ATAACGGCGTTCATTCTTGG	*hp0610*	FP of *hp0610*
hp0610-R	GCGGTTGTTATGCAAGGTTT	*hp0610*	RP of *hp0610*
omp6-F	GCCCGATTCTAAAGGGTTTC	*omp6*	FP of *omp6*
omp6-R	GGCCAAACTCTTTGGTGGTA	*omp6*	RP of *omp6*
hpn-F	ATGGCACACCATGAAGAACA	*hpn*	FP of *hpn*
hpn-R	GATGAGAGCTGTGGTGGTGA	*hpn*	RP of *hpn*
HP0256-QF	GCGCGCCCATGG AAAAATTCGCTTCTGTATTGG	*hp0256*	FP of *hp0256*
HP0256-QR	GCGCGCGGATCC TTACGCATGCAAGCCCTCTTT	*hp0256*	RP of *hp0256*

FP, forward primer; RP, reverse primer.

### Molecular cloning

Standard procedures were employed for plasmid cloning experiments in *E. coli *[49]. For complementation of a *Salmonella fliJ *mutant (strain MKM40, kind gift from the late Prof. R. M. Macnab), the HP0256 gene was amplified with primer pairs HP0256-QF/HP0256-QR (Table 4). The amplicons were digested with NcoI and BamHI, and ligated to similarly restricted pQE-60. *Salmonella *was transformed by electroporation using a standard protocol [50]. Electrocompetent *Salmonella fliJ *mutant cells were then transformed and transformants were selected on kanamycin (50 μg/ml). For complementation of the HP0256 mutant, a full length copy of the gene was introduced into the HP0203-HP0204 chromosomal intergenic region of a P79 HP0256-KO mutant according to the method described by Langford *et al*. using the pIR203K04 plasmid [51]. As expression of HP0256 is controlled by a promoter further upstream in a 5-gene operon, the gene was first amplified using the primers HP0256-F2 and HP0256-R and fused to the *flaA *promoter amplified using the primers FLA-F2 and FLA-R2, by overlap extension PCR. This composite fragment *flaA *promoter-HP0256 was then cloned into pIR203K04 as a *Cla1/BamH1 *fragment.

### Transmission electron microscopy

Cell samples were subjected to negative staining. Whole cells of *H. pylori *were grown on a plate containing brain heart infusion (BHI) supplemented with 10% foetal calf serum, for 24 h in a micro-aerobic atmosphere. Next, cells were harvested and carefully resuspended in 2% ammonium molybdate (Sigma) with 70 μg/ml bacitracin (Sigma), as a wetting agent. 5 μl cell preparation was applied to a copper grid overlaid with a carbon-coated Formvar film. The excess sample was carefully removed and the copper grid was dried. The copper grids were observed in a JEOL JEM-1200EX transmission electron microscope at an accelerating voltage of 80 kV.

### Plate motility assay

*H. pylori *strains and mutants were grown for 2 days on CBA plates and then stab inoculated on Brucella soft agar plates containing 0.3% (w/v) agar and 5% (v/v) heat-inactivated foetal bovine serum (Sigma). Motility plates were incubated at 37°C in an atmosphere containing 5% CO_2 _and periodically observed for halo formation.

### Protein electrophoresis and blotting

A standard protocol was used to perform sodium dodecyl sulfate-polyacrylamide gel electrophoresis [52] and immunoblotting. Proteins from 12.5% acrylamide gels were transferred onto nitrocellulose membrane by electroblotting [53]. Polyclonal antibody directed against *H. pylori *flagellin and hook protein was used as primary antibody [33]. Anti-rabbit antibody conjugated to horseradish-peroxidase (Sigma) was used as secondary antibody. Hydrogen peroxide and 4-chloro-1-naphtol (Sigma) were employed for colour development.

### Microarray analysis

To compare the transcriptional profiles of the wild-type and HP0256 mutant strains, a *H. pylori *whole genome microarray was used in a Common Reference or Type II experimental design whereby Cy5-labelled RNA from each strain was co-hybridised to an array with a Cy3-labelled genomic DNA reference. The microarray experiment was performed as described previously by Douillard *et al*. [54]. Four biological replicates, including a dye-swap, were performed for the global transcript comparison of the wild-type and the HP0256 mutant. The array design is available in BμG@Sbase (Accession No. A-BUGS-18; http://bugs.sgul.ac.uk/A-BUGS-18) and also ArrayExpress (Accession No. A-BUGS-18). Fully annotated microarray data have been deposited in BμG@Sbase (accession number E-BUGS-98; http://bugs.sgul.ac.uk/E-BUGS-98) and also ArrayExpress (accession number E-BUGS-98).

### Quantitative analysis of transcription by Real-Time PCR

Quantitative real-time PCR (qRT-PCR) was performed as a confirmatory test on selected genes following global transcript analysis by microarray. Real-time PCR primers were designed using the Primer3 software package [55] and are listed in Table 4. qRT-PCRs were performed as previously described [54]. Reactions were performed in triplicate (technical replicates) from at least two independent RNA preparations (biological replicates).

### Adhesion and interleukin-8 ELISA

AGS gastric epithelial cells were grown in six-well plates at 3.2 × 10^5 ^cells per well for six days. *H. pylori *cells were harvested from two-day old plate cultures of wild-type strain or the HP0256 mutant using 1 ml sterile PBS. Bacteria were washed twice with HAMS F12 media (Sigma, UK) and adjusted to an OD_600 _of 0.3. Cells were added to three wells of pre-grown AGS cells at a multiplicity of infection of 500:1 and incubated at 37°C and 5% CO_2 _for 3 h. Next, 1 ml of medium was removed and stored at -20°C for ELISA analysis. Cell supernatants were tested for IL-8 protein using the commercially available DuoSet ELISA kit (R and D Systems, Minneapolis, MN) as per manufacturer's instructions.

An *H. pylori *adhesion assay was performed to measure bacterial cells adhering to the AGS monolayer [56]. The remaining medium was discarded and the AGS cells were washed three times with room temperature HAMS F12 media. AGS cells were then treated with 1 ml of 0.2 μM filter-sterilized saponin (Sigma) for 15 min at 37°C. Lysed cells were collected into a sterile 1.5 ml tube and centrifuged for 10 min at 13,000 rpm. The pellet was resuspended in 1 ml sterile PBS. Dilutions were prepared and plated in duplicate on CBA (Columbia base agar) plates. Controls were included to measure any differences in starting numbers of bacteria between strains. *H. pylori *colonies were counted after 48 h and averaged. Adhesion of the HP0256 mutant was expressed as a percentage of the wild-type. Experiments were performed in triplicate.

## Authors' contributions

FPD participated in the generation of HP0256 mutants in two distinct *H. pylori *type strains, participated in the transmission electron microscopy, performed protein electrophoresis and immunoblotting analyses, global transcript analysis, quantitative analysis of transcription by quantitative Real-time PCR, participated in motility plate assay and drafted the manuscript. KAR performed adhesion assay, participated in the generation of HP0256 mutants in two distinct *H. pylori *type strains, immunoblotting analyses, complementation of *Salmonella *FliJ mutant, motility plate assay, performed interleukin-8 ELISA and drafted the manuscript. MCL participated in the generation of HP0256 mutants in *H. pylori *type strains. DLC participated in complementation of *Salmonella *FliJ mutant. SAM performed bioinformatics analyses, participated in its design and coordination and helped to draft the manuscript. CWP performed transmission electron microscopy. JH designed and produced the microarrays, conceived the transcriptome experimental design, and helped analyze the array data. POT conceived the study, and participated in its design and coordination and drafted the manuscript. All authors read and approved the final manuscript.
